# Factors associated with moderate neonatal hyperthyrotropinemia

**DOI:** 10.1371/journal.pone.0220040

**Published:** 2019-07-18

**Authors:** Ernesto Cortés-Castell, Mercedes Juste, Antonio Palazón-Bru, Mercedes Goicoechea, Vicente Francisco Gil-Guillén, María Mercedes Rizo-Baeza

**Affiliations:** 1 Department of Pharmacology, Pediatrics and Organic Chemistry, Miguel Hernández University, San Juan de Alicante, Alicante, Spain; 2 Department of Clinical Medicine, Miguel Hernández University, San Juan de Alicante, Alicante, Spain; 3 Center for Advanced Research in Public Health, Generalitat Valenciana, Valencia, Valencia, Spain; 4 Department of Nursing, University of Alicante, San Vicente del Raspeig, Alicante, Spain; Tabriz University of Medical Sciences, ISLAMIC REPUBLIC OF IRAN

## Abstract

**Background:**

Maternal iodine deficiency is related to high neonatal thyroid-stimulating hormone (TSH) values, with the threshold of 5 mIU/L recommended as an indicator of iodine nutrition status. The objective of this study was to analyse possible risk factors for increased TSH that could distort its validity as a marker of iodine status. The clinical relevance of this research question is that if the factors associated with iodine deficiency are known, iodine supplementation can be introduced in risk groups, both during pregnancy and in newborns.

**Methods:**

A case-control study was carried out in a sample of 46,622 newborns in 2002–2015 in Spain. Of these, 45,326 had a neonatal TSH value ≥5 mIU/L. The main variable was having TSH ≥5 mIU/L and the secondary variables were: sex, gestational age, day of sample extraction and maternal origin. Associated factors were analysed through a logistic regression model, calculating the odds ratio (OR).

**Results:**

The factors associated with this outcome were: male sex (OR = 1.34, 95% CI: 1.20–1.50, p<0.001), originating from an Asian/Oceanic country (OR = 0.80, 95% CI: 0.54–1.20, p = 0.536) or Europe (OR = 0.80, 95% CI: 0.66–0.96, p = 0.285) (including Spain, OR = 1) [p<0.001 for America (OR = 0.54, 95% CI: 0.44–0.68) and p = 0.025 for Africa (OR = 0.78, 95% CI: 0.62–0.97)] and fewer days from birth to sampling (OR = 0.80, 95% CI: 0.77–0.82, p<0.001).

**Conclusions:**

The risk of high neonatal TSH without congenital hypothyroidism is higher in males, decreases with a greater number of days from birth to extraction, and is dependent on maternal ethnicity but not on gestational age.

## Introduction

In newborns without hypothyroidism, several factors have been found to influence thyroid-stimulating hormone (TSH) values, classified in the following groups: maternal, pregnancy, childbirth and neonatal [1–4]. For those factors related to the newborn, the most important is low birth weight [5]. There is also strong evidence that deficient maternal iodine values due to nutritional status are associated with high TSH values (≥5 mIU/L) [6–9]. This threshold has been used in the scientific literature as an indicator of the iodine nutrition status in a population measured through neonatal screening and its evolution over time [10–14], as well as the effect of measures to supplement the diet with iodine [15–18]. For this reason, this threshold has been recommended for population analysis, excluding cases of severe maternal or foetal iodine deficiency [19]. However, at present, there is discussion about adapting the threshold according to different situations of the newborn, such as the time of year, weight of the newborn, or days from birth to blood sampling [20–22]. If certain factors influence the neonatal TSH values, it is not logical to use a single cut-off point to evaluate the nutritional status of the mother. This cut-off point would need to be adapted to the different circumstances by which it can be modified, such as gestational age and the sex of the newborn. Moreover, measurement of the neonatal TSH can only orient towards a maternal iodine deficit, though it is nevertheless an inexpensive way to obtain this information as no additional test is needed apart from the almost universal neonatal screening for hypothyroidism. Accordingly, the present study examined the importance of certain parameters (sex, gestational age, birth weight, days from birth to blood sampling, and maternal origin) as potential risk factors associated with the presence of TSH values ≥5 mIU/L in newborns, in order for the neonatal TSH values to be relatively valid in the determination of the nutritional iodine status of the mothers and, consequently, in the general population.

This is an important issue as the identification of these factors could be used to introduce iodine supplementation during pregnancy in risk groups. In addition, this could be taken into account in newborns with abnormal TSH values according to risk groups.

## Materials and methods

### Study population

The study population comprised newborns from the Valencian Community (Spain) who underwent neonatal screening according to the "Neonatal screening programme for congenital diseases" [23], which is provided free of charge and with universal coverage. This programme is centralised for the whole of the Valencian Community, with samples of heel blood being collected from newborns at each of the maternal hospitals in the Community prior to hospital discharge.

### Study design and participants

This was a case-control study of newborns in the Valencian Community between 2002 and 2015 (n = 649,849) who had TSH values ≥5 mIU/L (n = 52,184) in neonatal congenital hypothyroidism screening. We excluded those neonates who were positive for congenital hypothyroidism, and those whose samples did not meet the pre-analysis quality criteria [adequate blood volume, uniformity of the application on paper, degree of saturation, no exposure to interfering agents (alcohol, water, etc.), incorrect drying, excessive heating and reaching the laboratory within 15 days (n = 6,845)] [23]. A random sample of all infants with normal TSH (TSH <5 mIU/L) (n = 1,296) who were selected using screening software was used as the control group.

### Variables and measurements

The primary variable was the presence of a TSH value ≥5 mIU/L. Sex, gestational age, the day of peripheral blood sampling for neonatal hypothyroidism screening, and maternal origin were used as secondary variables. The subgroups formed with the secondary variables were defined as: i) preterm (gestational age <37 weeks) [15]; ii) maternal origin, i.e. Spain, North Africa, sub-Saharan Africa, Latin America, the rest of Europe and Asia; and iii) sex (male or female). All variables were obtained from the neonatal screening report.

Quantification of TSH was performed with the AutoDELFIA Neonatal hTSH kit from Perkin Elmer using a TSH cut-off value of 7.5 mIU/L, as per the majority of the Spanish laboratories performing this test according to the Spanish Neonatal Screening Association criteria [23]. The intra- and inter-assay cut-off values were 7.0% and 8.0%, and the sensitivity limit was 0.2 mIU/L. To ensure correct measurement, before each assay an internal quality control must be passed, using the controls provided by the AutoDELFIA Neonatal hTSH kit. Required external quality assurance controls include a monthly Spanish Neonatal Screening Association quality assessment in neonatal early detection and a quarterly assessment by the Newborn Screening Quality Assurance Program of the Centers for Disease Control. Regarding the reliability of the TSH measurements, both internal quality control (PerkinElmer, Life Sciences, Delfia Neonatal hTSH) and external control programs "Infant Screening Quality Assurance Program" (Center for Disease Control, Atlanta, USA) and also the Asociación Española de Cribado Neonatal [Spanish Association of Neonatal Screening] were used. The intra- and inter-assay variation results were <10% for a reference value of around 15 mIU/l. This is a key point to obtain relevant results for clinical practice [24,25].

### Sample size calculation

No sample calculation was performed beforehand, as the total database of positive cases was available. Consequently, we determined the power to contrast an odds ratio (OR) different from 1 (null effect between the exposure and the outcome). For this calculation, we used the sex of the child as a factor and the following parameters: type I error of 5%, expected proportion of TSH values ≥5 mIU/L (0.07 for males and 0.05 for females) and our sample size (27,099 males and 19,523 females) [22]. With these parameters, the power of the contrast was close to 100% with the sample collected. This was due to the fact that the type II error was very small (<0.001).

### Statistical analysis

We performed multiple imputations for the missing data in our database using chained equations imputation. This was done because it is the recommended method to manage missing data, completing missing observations with plausible estimated values derived from the analysis of the data that are available. This first requires corroborating that the data are not distributed as missing completely at random (MCAR) [26]. This was done performing Little’s MCAR test (p<0.001). The variables were described using absolute and relative frequencies for the qualitative variables, whilst the mean and standard deviation were used to describe the quantitative variables. To determine differences between cases and controls, Pearson's Chi-square and Student's *t*-tests were performed. The multivariate analysis (logistic regression model) included all the secondary variables, with TSH ≥5 mIU/L as the dependent variable. With this model we obtained the adjusted odds ratios (OR) for all the analysed factors. The likelihood ratio test was used to compare the constructed model with the null model (goodness of fit). The Type I error was set at 5% and for each relevant parameter its associated confidence interval (CI) was calculated. All calculations were performed with IBM SPSS Statistics 25.

### Ethical considerations

The study was adapted to the ethical principles of the Declaration of Helsinki and was approved by the Ethics Committee for Clinical Research of the Directorate General of Public Health and Center for Advanced Research in Public Health of the Valencian Community (code ACN). Informed consent of the parent or guardian of the newborn was not required, in compliance with the current legislation in medical ethics. Moreover, the data, which are data from routine clinical practice in neonatal screening, were anonymized and encrypted, satisfying the data protection law.

## Results

We had the following missing data for our variables: 2,359 for sex (5.06%), 1,646 for gestational age (3.53%) and 4,267 for maternal origin (9.15%). These were imputed using chained equations imputation.

The incidence of TSH ≥5 mIU/L in the total number of newborns was 7.52% (95% CI: 7.28–7.77%). When we compared the two groups (Table 1), we found that amongst those newborns with a TSH ≥5 mIU/L there was a higher proportion of males (58.3 vs 51.0%, p<0.001), a higher mean gestational age (38.9 vs 38.8 weeks, p = 0.016), a lower mean number of days from birth to extraction (2.0 vs 2.6 days, p<0.001) and differences in maternal origin (p<0.001).

**Table 1:** Analysis of subclinical hypothyroidism in the Valencian Community (Spain).

	TSH ≥5 mIU/L	TSH <5 mIU/L				
	n = 45,326	n = 1,296				
Variable	n(%)/x±s	n(%)/x±s	p	Adj. OR	95% CI	p
**Male sex**	26,438(58.3)	661(51.0)	<0.001	1.34	1.20–1.50	<0.001
**Gestational age (w)**	38.9±1.7	38.8±1.8	0.016	1.01	0.98–1.04	0.536
**Place of origin:**						
**Spain**	36,096(79.6)	962(74.2)	<0.001	1	N/A	N/A
**Africa**	2,662(5.9)	91(7.0)		0.78	0.62–0.97	0.025
**America**	1,876(4.1)	92(7.1)		0.54	0.44–0.68	<0.001
**Rest of Europe**	3,947(8.7)	125(9.6)		0.80	0.66–0.96	0.285
**Asia/Oceania**	745(1.6)	26(2.0)		0.80	0.54–1.20	0.536
**Days from birth to extraction**	2.0±1.3	2.6±1.3	<0.001	0.80	0.77–0.82	<0.001

Abbreviations: Adj. OR: adjusted odds ratio; CI: confidence interval; n(%), absolute frequency (relative frequency); N/A, not applicable; TSH, thyroid-stimulating hormone; x±s, mean ± standard deviation. ORs were adjusted for: infant’s sex, gestational age, maternal origin and days from birth to extraction. Goodness-of-fit of the model: Χ^2^ = 238.36, p<0.001.

The results obtained were confirmed through multivariate analysis. The adjusted OR for the male neonates was 1.34 (95% CI: 1.20–1.50, p<0.001). The OR for the maternal origins of Africa (OR = 0.78, 95% CI: 0.62–0.97, p = 0.025) and America (OR = 0.54, 95% CI: 0.44–0.68, p<0.001) was less than one, and also decreased with the number of days from birth to sample collection, with an OR of 0.80 for each additional day of age (95% CI: 0.77–0.82, p<0.001). The model had a satisfactory fit (p<0.001 in the likelihood ratio test).

## Discussion

In the Valencian Community, according to the data from 2002–2015, the neonatal incidence of TSH ≥5 mIU/L was 7.52%. However, this percentage is very likely overestimated given that that the number of days from birth to blood collection has a great influence. These data are important, as it should be borne in mind that a high percentage of the 9% of newborns with TSH values between 5–10 mIU/L may eventually present congenital hypothyroidism and it is, therefore, important to establish a cut-off point and a fixed day of age at sampling, or changing values according to days from birth to sample collection [27–29].

Contrary to what might be expected, neonates born to mothers of Spanish origin, who experience better economic conditions and access to a universal and free health system in Spain, have a higher risk of having high TSH. Finally, it was also found that male neonates have a higher risk of elevated TSH than female neonates [29].

It is difficult to compare the incidence with other populations because of the factors involved, the most important of which is the age at blood sampling, already described by others [20–22], with considerable variations between Spanish autonomous communities [30]. Another important factor for comparison is whether or not iodine antiseptics are used during childbirth and the first few days of life, which strongly affect TSH values [31], although this is not applicable in our case, as the conditions established in our maternity and neonatal units include a ban on the use of these types of antiseptics [23].

In the multivariate analysis, no clear differences were found between gestational age and the proportion of newborns with TSH values ≥5 mIU/L, whereas the bivariate analysis did reveal differences. These results are in contradiction with the literature reporting the highest incidence amongst infants with a low birth weight [5], but in agreement with what we have previously established regarding TSH values in preterm infants with low or adequate weight for their gestational age, in which the greatest quantitative importance results from the influence of the number of days from birth to sampling [2]. For these reasons, the interpretation of preterm values can lead to errors in screening. Therefore, it is advisable in screening programs to repeat the tests in these children at 40 weeks post-conception [23,32].

The influence of ethnic origin is clear. It has been widely described, most recently in the Balkans [33]. In our case, Spanish mothers are more likely to have a newborn with high TSH values, and given that the health system offers the same services to the entire population and that there is a globalisation of eating habits, ethnicity must be a factor in this higher incidence.

Concerning the higher percentage amongst male newborns with elevated but not pathological TSH, this is in contrast with the higher number of female newborns with congenital hypothyroidism (3/1) in our region and in general [22,23]. These elevated non-pathological TSH values among male newborns are in line with what we found in preterm infants [2] and with what has been documented by others in children and which increase in adolescents and adults [34,35].

We did not consider the time of year, unlike other studies [1], because we demonstrated previously that in our environment the differences in average monthly temperatures have a range of about 18±6°C [32], and these fluctuations do not interfere with measurements that are very sensitive to seasonal temperature changes in neonates such as immunoreactive trypsin [36,37]. Other factors such as the smoking behaviour of the mother have been studied by others with no impact shown on neonatal TSH levels [1]. The implications of the results of this study are mainly the great variability in the proportion of abnormal TSH values according to the days elapsed from birth to sampling. At present, the 5 mIU/L threshold to determine the nutritional iodine deficit in pregnant women is a fixed value which only specifies that the sample should be taken with effect from 48 hours after birth. We believe this presents an important limitation regarding the recommended value. To address this problem, the cut-off point should be modified according to the day of blood extraction or, although more difficult for reasons of sampling strategy, the day of extraction should be fixed for all neonatal screening to eliminate the great variability produced by this factor. This issue needs much greater study as it is very clinically relevant in neonatal screening.

### Strengths and limitations of the study

The main strength of this study is the large sample size, which yielded a very high statistical power and can be generalized to the entire newborn population. The multivariate analysis performed is another strength, as it provides the true influential value of the parameters analysed.

As limitations of the study, once the randomised extraction of cases of TSH <5 mIU/L was performed, they were found to be just a small proportion of all neonates with normal values, which could lead to bias. However, we have to take into account that our study was only designed to determine associated factors, with no outcome predictions. Another limitation is that no information was available on the length of time that immigrant mothers had been in Spain or whether these women had adapted to the health and eating habits. Therefore, we cannot conclude that they had a very different diet or that ethnic reasons led to this lower risk [38,39]. Another limitation of this study is that measurement of neonatal TSH levels is not totally sensitive for the nutritional iodine status of the mother. Nevertheless, we feel it can be used as a good approach, bearing in mind that no extra cost is incurred with the use of neonatal screening for congenital hypothyroidism. It can therefore serve as an indication prior to recommending a urine iodine test for the mother. Finally, other factors not contemplated in this study might exist, the addition of which to the logistic regression model would give the results greater validity. This remains for future studies.

## Conclusion

The risk of a newborn having a high TSH value without congenital hypothyroidism is higher in males, decreases with the number of days from birth to sampling, and depends on maternal ethnicity. This risk, however, does not depend on gestational age. As a clinical implication, based on our results, the TSH cut-off point should be modified taking into account the day of blood extraction, the sex of the newborn, and the maternal origin.

## Supporting information

S1 Dataset Database of our study. (XLSX)
